# Hydrothermal Carbonization of Microalgae Biomass from Wastewater Treatment: Effects of Acid Pretreatment

**DOI:** 10.1021/acsomega.5c02582

**Published:** 2025-07-21

**Authors:** Adriana Paulo de Sousa Oliveira, Paula Peixoto Assemany, Castro Jackeline de Siqueira, Maurino Magno de Jesus Júnior, Fábio de Ávila Rodrigues, Luiz Fernando Cappa de Oliveira, Mariana Toledo Clemente Campos, Angélica de Cássia Oliveira Carneiro, Maria Lúcia Calijuri

**Affiliations:** † Department of Civil Engineering, 28120 Federal University of Viçosa, Viçosa, Minas Gerais 36570-900, Brazil; ‡ Department of Environmental Engineering, Federal University of Lavras, Lavras, Minas Gerais 37200-900, Brazil; § Department of Chemistry, Federal University of Viçosa, Viçosa 36570-900, Minas Gerais, Brazil; ∥ Department of Chemistry, 28113 Federal University of Juiz de Fora, Juiz de Fora, Minas Gerais 36036-900, Brazil; ⊥ Department of Forest Engineering, Federal University of Viçosa, Viçosa, Minas Gerais 36570-900, Brazil

## Abstract

Hydrothermal carbonization (HTC) of wastewater-grown microalgae biomass is an attractive route for producing hydrochar (a solid fuel). However, HTC optimizations are needed to enhance energy recovery from biomass. Among the possible strategies, biomass pretreatment through acid washing is often applied to promote increased carbon content. The effects of this pretreatment require additional research, as the biomass may undergo adverse changes and affect the characteristics of the hydrochar. Therefore, in this study, three biomass samples grown in swine wastewater were washed with HCl solution and then subjected to HTC at 170 °C for 10 min. The pretreatment removed ash content from 15.9–38.6% in the raw biomass to 9.8–10.7% in the acid-washed biomass. This reduction was accompanied by a twofold increase in protein content. During HTC, acid pretreatment contributed to the formation of semicrystalline structures. It resulted in a hydrochar with a higher heating value (up to 22.0 MJ/kg) and energy recovery (up to 68.2%) compared to hydrochar without pretreatment (up to 13.1 MJ/kg and 57.2%, respectively). Despite the positive aspects, the HTC of acid-pretreated biomass increased the N and S contents of the hydrochar (61.4–70.7 g·N/kg and 5.9–6.5 g·S/kg) when compared to the hydrochars derived from biomass without acid washing (33.7–46.5 g·N/kg and 4.5–4.6 g·S/kg), which poses a risk of greenhouse gas emissions if used as fuel. Thus, the beneficial and undesirable effects of acid washing as a pretreatment of algal biomass for biofuel production must be weighed for each case with an integrated analysis of the entire process. Life cycle and techno-economic analyses—especially when considering scale-up—can be used for this purpose by providing feasibility results in real microalgae bioenergy contexts.

## Graphical abstract

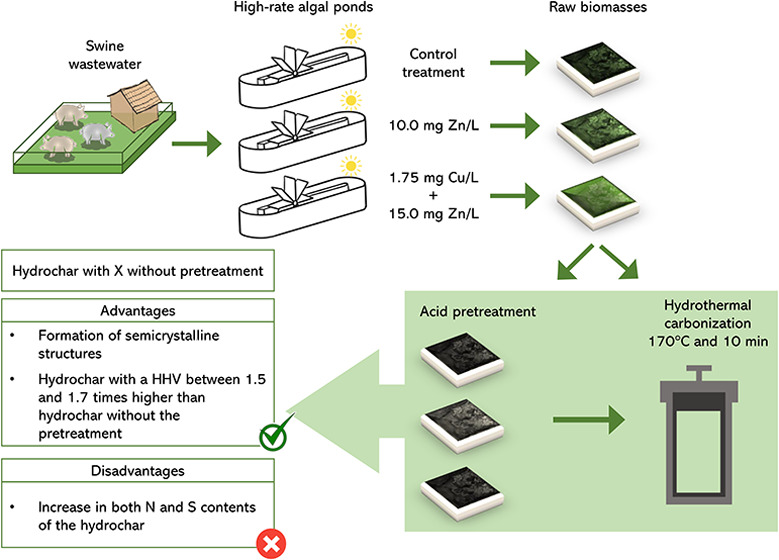

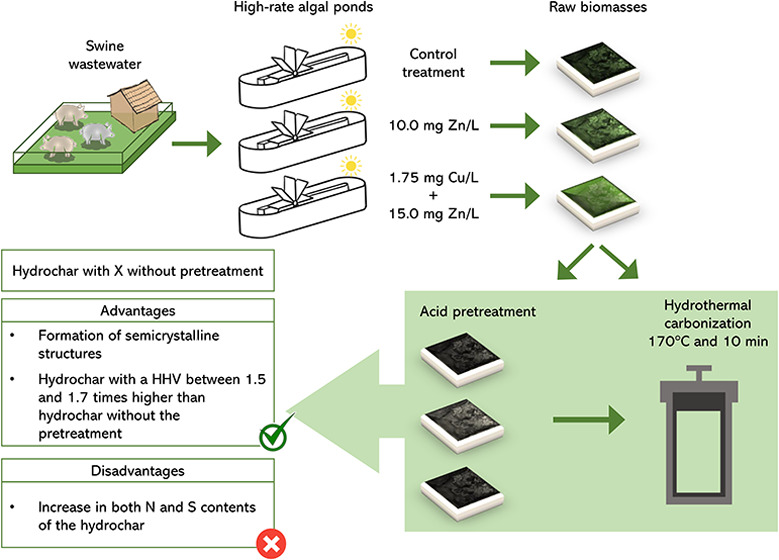

## 1 Introduction

The growing energy demand, combined with the severe depletion of fossil fuels and environmental pollution, raises significant global concerns. New technologies need to be developed to address these challenges. In this context, microalgae biotechnology stands out as an innovative solution crucial to achieving the UN’s Sustainable Development Goals. This capability is due to the versatility of microalgae, which can grow in wastewater, allowing for the combination of effluent treatment with the production of algal biomass that can be converted into bioenergy.^1,2^ This combination is a versatile and holistic solution to emerging environmental challenges.^3^

However, significant efforts are needed to optimize this biotechnology and establish an efficient process for producing bioenergy from microalgae. Among the challenges, the composition of the produced biomass can be highlighted. In addition to value-added products such as proteins, lipids, and carbohydrates, this biomass is also characterized by high moisture (92–99%) content observed even after the harvesting step.^4,5^ Therefore, it is necessary to process large amounts of water to obtain microalgae bioproducts,^6,7^ which may increase bioenergy production costs.

To overcome the challenge related to water content in the harvested biomass, previous studies investigated thermal processes that use wet biomass, such as hydrothermal gasification, liquefaction, and carbonization. Among these, hydrothermal carbonization (HTC) proved promising results since a raw material with 75% and 90% moisture content may be suitable for the process, reducing the need for algal biomass concentration.^8,9^ Furthermore, it has lower pressure and temperature requirements (>2 MPa and 180–220 °C) compared to liquefaction (5–20 MPa, 280–370 °C) and gasification (22–36 MPa and 400–500 °C). Also, reaction time is generally less than 90 min, with having low energy demand compared to other hydrothermal processes.^10^

Although the chemistry of HTC is still not fully understood,^11^ this process alters the original structure of biomass through chemical reactions such as hydrolysis, dehydration, decarboxylation, aromatization and recondensation.^12^ The result is the formation of a gaseous and aqueous phase, and a solid material named hydrochar. Hydrochar has several benefits over its original biomass: higher carbon content, more oxygenated functional groups, higher energy density, and lower aromatization degree.^12^ HTC and Co-HTC processes, although dealing with low-cost waste, mimic nature to quickly provide a solid material that would otherwise take millions of years to form naturally.^13^ Given these advantages, HTC has been applied in several studies, enabling the conversion of algal biomass into hydrochar. This carbon-rich material can be used as solid fuel, fertilizer, and adsorbent.^14,15^

Therefore, HTC is suitable for processing algal biomass grown in wastewater and overcoming the challenge of high moisture. However, the challenges are not limited to this, another unattractive characteristic of algal biomass grown in wastewater is the high ash content, between 15 and 60%, depending on the characteristics of the effluent, the cultivation conditions, and the harvesting technique used,^4,5,16^ Ash is undesirable in a solid fuel because it reduces the yield of the converting biomass into biofuel process, compromises the quality of the final products, offers the risk of damaging equipment, and contributes to slagging production and fouling.^6,17^ For these reasons, some studies have evaluated biomass pretreatments to reduce ash content.

For example, a pretreatment of microalgae biomass grown in domestic sewage with an acid solution (4 M HCl) promoted a reduction in ash content from 44.7% to 14.5%, with a hydrochar reaching a higher heating value (HHV) of 26.6 MJ/kg.^18^ Hydrochar from microalgae biomass grown in swine wastewater presented an HHV equal to 18.6 MJ/kg. This value increased 1.37 times after the hydrochar washing with an acid solution (1 M HCl) to reduce ash and other byproducts.^19^ A 90% of ash removal efficiency was achieved by Wang and collaborators,^20^ who performed a biomass acid washing with HCl 5%, promoting an increase in the HHV of 1.16 times.

Acid washing of biomass is a simple process that uses readily available materials and does not require specific equipment. Different researchers,^18,19,21^ have used this alternative in conjunction with HTC to improve the yield and characteristics of hydrochar. Although acid washing provides promising results in ash removal, this methodology should be further investigated to explain its relationship with increases in HHV. Acid washing is not restricted to ash removal and can cause adverse changes in the biomass, affect the composition of the hydrochar, and interfere with its use. Identifying these changes regarding the structure and chemical profiles in biomass and hydrochar is essential to enhance the production of this solid fuel.

Several articles on optimizing the HTC process suggest optimum temperature conditions, reaction time, and solid–liquid ratio to maximize the hydrochar yield.^22^ This study presents another possibility for optimizing HTC by investigating the scope and limitations associated with acid washing pretreatment. More specifically, the objectives of this study were to apply an acid solution as a biomass HTC pretreatment and to evaluate the interference of the pretreatment in the performance of the HTC and the characteristics of the hydrochar. By trying to better explore the role of acid washing in the HTC process, this manuscript overcomes some limitations of the HTC from wastewater-grown microalgae biomass.

It focuses on improving the energy process yield and hydrochar quality. Also, by making the process more efficient and profitable, with technologies that minimize operating costs and maximize resource recovery, environmental impacts, such as water pollution and greenhouse gas emissions, can be reduced. The valorization of sanitation waste also contributes to the circular economy, promoting a sustainable cycle that integrates agricultural and waste management practices and stimulating technological innovations for a more sustainable sector.

In this context, this study investigates the potential of acid washing as a pretreatment strategy for microalgal biomass subjected to HTC. The hypothesis is that acid washing can enhance hydrochar quality for use as solid biofuel. To test this, hydrochars produced from pretreated and untreated biomass were evaluated based on key performance indicators metrics, including ash content, HHV, energy yield, carbon retention, and nitrogen and sulfur concentrations—parameters directly linked to combustion performance and environmental impact.

## 2 Materials and Methods

### 2.1 HTC Substrates

Biomass was produced in three pilot-scale high-rate algal ponds (HRAPs) installed outdoors and operated with swine wastewater (SW). SW presented chemical oxygen demand equal to 436.8 mg/L; total Kjeldahl nitrogen (TKN) of 595.9 mg/L; total phosphorus of 25.8 mg/L, total copper equal to 0.1 mg/L, and total zinc of 0.5 mg/L (quantified according to APHA.^23^ Each HRAP received SW with the same macronutrient constitution. However, in each HRAP, different concentrations of Cu and Zn were adjusted, as a previous study showed that SW with adjustment in these elements favors biomass productivity.^24^

Therefore, two HRAPs were operated with SW with the adequacy of Cu and Zn at concentrations of 10 mg Zn/L and 1.75 mg Cu/L + 15.0 mg Zn/L. The third HRAP was used as a control with the Cu and Zn concentrations initially observed in SW (0.1 mg Cu/L + 0.5 mg Zn/L). All three HRAPs were operated in batch mode for a period that varied from 20 to 25 days according to the decrease in chlorophyll *a* used as a monitoring parameter. At the end of the cultivation, the biomass was harvested through the pH adjustment with NaOH addition and then frozen and lyophilized. In this study, the name biomass 1 (B1) refers to the control treatment; biomass 2 (B2) was cultivated in SW with 10 mg Zn/L; and biomass 3 (B3) was developed in SW with 1.75 mg Cu/L + 15.0 mg Zn/L. All biomass samples presented a mixed composition, including microalgae of the genus *Chlorella* sp., bacteria, other microorganisms, and organic and inorganic matter. Each of the three biomass samples was divided into two portions: one was subjected to pretreatment (described in item 2.2) followed by HTC (described in item 2.3), while the other was used directly in HTC.

### 2.2 Pretreatment

Biomass washing with a 0.25 M HCl solution was adopted as a pretreatment for the HTC because it maintained the highest carbon content in the biomass while demonstrating the best performance in ash removal according to preliminary tests (Table S1). Biomass with particles between 1–1.4 mm was used (obtained using a Wiley mill), which was transferred to flasks containing HCl solution in a proportion of 1:80 (m/v).^25^ The flasks were placed in an incubator with orbital shaking at 120 rpm^26^ for 1 h. In the sequence, the biomass was washed with distilled water until the supernatant had a pH close to 7.0, i.e., this was the final pH after pretreatment. Then, it was dried in an oven at 40 °C until constant weight. Biomass before and after the pretreatment (washed biomass—WB) was characterized and used in HTC tests.

### 2.3 Hydrothermal Carbonization

HTC was conducted in a Parr 5500 Series compact reactor with a capacity of 300 mL and with a temperature and rotation speed controller. Biomasses with and without pretreatment were transferred to the reactor with distilled water in a ratio of 1:15.^18^ During the acid wash, solids were lost due to solubilization. To maintain the same proportion of water and biomass during HTC, the tests were conducted considering the mass of solids at the reactor inlet. In other words, the same amount of raw and pretreated biomass was added. The reactor was purged with nitrogen gas for 5 min and kept under stirring at 500 rpm at 170 °C for 10 min.^8^ After the reaction time, the reactor was cooled using an internal system that allowed ice water recirculation. Then, the sample was removed from the reactor and centrifuged to separate the solid fraction (hydrochar), which was dried at 40 °C until constant weight.

The operating conditions of the reactor adopted in the present study were established in a previous study using algal biomass cultivated in wastewater. It was verified through a surface model that the energy retention in the hydrochar from that type of biomass decreased as the reaction time increased.^8^ However, the operating conditions used in the present study can be considered initial HTC conditions (early stage HTC). The hydrochars obtained with the application of pretreatment followed by HTC were named H-WB1, H-WB2, and H-WB3, associated with biomass B1, B2, and B3, respectively, after acid washing. The hydrochars produced only in HTC (without pretreatment) were named H-B1, H-B2, and H-B3, referring to the products derived from biomass B1, B2, and B3, respectively. All HTC tests were performed in triplicate. The results that characterized the hydrochar, referring to solid yield, energy yield, carbon yield, HHV, and nutrients N and S (described in item 2.4), were submitted to analysis of variance and the significance test at the level of 5% using the Minitab 17 software.

### 2.4 Characterization of Biomass and Hydrochar

The concentrations of macro and micronutrients were quantified in raw and pretreated biomass (WB), and the respective hydrochars (H-Bs and H-WBs) according to EMBRAPA.^27^ Phosphorus (P) was quantified by colorimetry; potassium (K) by flame photometry; calcium (Ca) and magnesium (Mg) by atomic absorption; sulfur (S) by turbidimetry; manganese (Mn), copper (Cu), cadmium (Cd), iron (Fe) and zinc (Zn) metals were determined in samples submitted to a digestion step followed by quantification by atomic absorption (Optima 8300, Waltham, USA). Nitrogen (N) concentrations were also determined using the Kjeldahl method. In addition, the concentrations of proteins and total lipids were quantified before and after the pretreatment.^24^

The performance of the HTC was evaluated by considering the characterization of the biomass and hydrochar. Ash content was determined through the mass difference before and after the sample combustion in a muffle furnace at 550 °C for 1h. HHV was obtained experimentally using the adiabatic bomb calorimetric method, and the energy yield calculation considered the solid yield (eqs 1 and 2):

1 $$\mathrm{Solid yield}\left(\%\right)=\frac{m_{\mathrm{char}}}{m_{\mathrm{biomass}}}\times 100$$

where *m*_char_ is the mass of hydrochar produced after HTC, and *m*_biomass_ is the amount of biomass subjected to HTC on a dry basis. In other words, *m*_biomass_ is the mass of raw material addition. It is important to highlight that solid yields in hydrochar from pretreated biomass were calculated based on the pretreated biomass.

2 $$\mathrm{Energy yield}\left(\%\right)=\mathrm{Solid yield}\frac{{\mathrm{HHV}}_{\mathrm{char}}}{{\mathrm{HHV}}_{\mathrm{biomass}}}$$

where HHV_char_ and HHV_biomass_ stand for the higher heating values of hydrochar produced after HTC and raw biomass, respectively.

Also, biomass and hydrochar were analyzed by determining C, H, and N contents using a PerkinElmer, PE-2400, series II elemental analyzer (Waltham, USA). The S content was obtained by turbidimetry, with reading in a spectrophotometer at a wavelength equal to 440 nm. The O content was determined from the difference between 100 and the sum of the ash, C, H, N, and S contents. The content of C was used in the calculation of C yield (eq 3). Additionally, the C, H, and O results were analyzed using the Van Krevelen diagram that presents the variations in O/C and H/C atomic ratios.

3 $$\mathrm{Carbon yield}\left(\%\right)=\mathrm{Solid yield}\frac{C_{\mathrm{char}}}{C_{biomass}}$$

where C_char_ and C_biomass_ stand for the carbon content on a dry basis of hydrochar produced after HTC and biomass added to the Parr reactor, respectively. Carbon yields in hydrochar from pretreated biomass were calculated based on the pretreated biomass.

Biomass and hydrochar were further characterized using X-ray diffraction (XRD) for the structural characterization and identification of the main compounds formed from HTC. For this, the diffraction system D8 Discover (Bruker, Billerica, USA), Cu-kα radiation (λ = 1.5418 Å) with an angular variation of 10–80° (2θ) was used. Scanning electron microscopy (SEM) (JEOL equipment model JSM-6010LA, Akishima, Japan) was used to evaluate the surface structure. Absorption spectroscopy in the infrared region with Fourier transform (FT-IR) was used to identify the chemical profiles using the Bruker Alpha FT-IR equipment (Billerica, USA), with 128 scans and 4 cm^–1^ of spectral resolution; samples were analyzed in ATR mode.

### 2.5 Characterization of the Aqueous Phase

The aqueous phase was characterized to identify the nutrient transfer from the biomass to liquid fraction and the effects of pretreatment. To this end, the P and total Kjeldahl nitrogen (TKN) contents were quantified in the aqueous phase according to APHA^23^ and total organic carbon (TOC) determined in the Shimadzu TOC 5000 equipment.

## 3 Results and Discussion

### 3.1 Characterization of Biomass

The three biomass used in the present study were cultivated in swine wastewater (SW) at different concentrations of Cu and Zn. The different cultivation conditions altered the composition of the biomass so that biomass B1 (biomass grown in the control treatment) presented the lowest ash and N content (Table 1). However, B1 contains the highest lipid, protein, and Pb concentrations. Biomass B2 and B3 (cultivated with adjustments in the Cu and Zn contents) presented higher concentrations of Cu and Zn, possibly resulting from algae assimilation and metal precipitation that occurred during cultivation and harvesting. Other constituents, such as the content of C, H, O, P, K, and HHV, remained similar in the three biomass samples.

**Table 1:** Biochemical Characterization of Biomass (on a Dry Biomass Basis) Used in Hydrothermal Carbonization Tests^a^

Parameter	B1	B2	B3	WB1	WB2	WB3
Proteins (%)	31.9	24.2	17.4	45.6	51.0	18.6
Total lipids (%)	17.0	2.6	9.0	11.3	11.1	7.4
Ash (%)	15.9	38.6	34.7	10.1	10.7	9.8
HHV (MJ/kg)	13.3	12.3	13.2	21.3	21.3	20.1
C (%)	28.6	27.3	29.8	42.8	44.5	45.1
H (%)	4.7	4.8	4.9	7.1	7.1	6.8
O (%)	17.0	18.3	16.8	27.7	27.0	20.4
N (g/kg)	33.7	41.6	46.5	70.7	66.1	61.4
P (g/kg)	40.0	43.5	42.5	4.0	3.6	3.9
K (g/kg)	15.5	12.3	16.6	0.3	0.3	0.3
Ca (g/kg)	60.7	75.1	68.9	5.7	8.7	5.4
Mg (g/kg)	9.0	7.6	4.7	0.3	0.3	0.3
S (g/kg)	4.6	4.5	4.5	6.2	6.5	5.9
Cu (g/kg)	0.3	2.2	2.8	0.3	2.0	0.3
Fe (g/kg)	3.9	3.0	2.2	4.7	4.7	4.3
Zn (g/kg)	3.8	20.5	27.7	0.4	0.3	0.4
Mn (g/kg)	0.4	0.3	0.2	0.0	0.1	0.0
Na (mg/kg)	1.1	1.1	1.5	0.0	0.1	0.0
Ni (mg/kg)	4.6	5.7	3.8	7.6	7.4	7.3
Pb (mg/kg)	33.0	17.4	25.4	2.8	7.9	2.4
Cr (mg/kg)	14.5	14.6	10.0	21.9	20.5	20.9

a B1, B2, B3 = raw biomass; WB1, WB2, WB3 = acid-washed biomass.

Comparing raw biomass (B1, B2, and B3) with the respective acid-washed biomass (WB1, WB2, and WB3), a considerable decrease was observed in P, K, Ca, Mg, Zn, Mn, Na, and Pb, with removals ranging between 55 and 100% in the pretreated biomass. Also, a 90% reduction of Cu was observed in WB3 biomass. These results can be attributed to ash removal, which promotes a decrease of ash content from 15.9% – 38.6% in the raw biomass to 9.8% – 10.7% in the acid-washed biomass. Consequently, a reduction in mass was observed in all biomass samples that received pretreatment. When comparing the solids content (on a dry basis) before and after acid washing, we observed a reduction of approximately 36%.

After the acid washing, variations in HHV were observed, changing from 12.3–13.3 MJ/kg before pretreatment to 20.1–21.3 MJ/kg after the pretreatment application. A similar result was reported in other studies. A reduction in the ash content of microalgae biomass from 27.1 to 13.5% resulted in an HHV increase from 24.4 to 26.2 MJ/kg.^6^ Also, a decrease in ash content from 44.7 to 14.4% was accompanied by an increase in HHV from 9.5 to 24.2 MJ/kg.^18^ The HHV improvement caused by a reduction in ash content can be explained with the equation obtained by Channiwala and Parikh,^28^ which relates the calculation of HHV for fuels based on elemental analysis: HHV = 0.3491C + 1.1783H + 0.1005S – 0.1034O – 0.0151N – 0.0211Ash (MJ/kg).

While the elements C, H, and S increase the HHV, the contents of O, N, and ash negatively affect this parameter. The ash does not participate in the combustion process, and high levels of this constituent represent a decrease in energy potential.^29^ When comparing the measured HHV values (Bs = 12.3–13.3 MJ/kg and WBs = 20.1–21.3 MJ/kg) with those calculated using the Channiwala and Parikh equation (Bs = 12.5–13.7 MJ/kg and WBs = 20.2–21.4 MJ/kg), it is noted that they are similar, demonstrating the consistency of the data.

Also, the reduction in ash led to a decrease in solids, which, in proportional terms, increased the content of C, H, and S elements in the pretreated materials, contributing to a higher HHV. Other analyzed parameters, i.e., O, N, Cr, and Ni, increased in the acid-washed biomass due to mass balance. Also, this reduction was accompanied by a 2 times increase in protein content, similar to those observed in previous works.^6,30^

### 3.2 HTC Performance

#### 3.2.1 Energy Characterization

The hydrochars obtained with the application of pretreatment followed by HTC were named H-WB1, H-WB2, and H-WB3, associated with biomass WB1, WB2, and WB3, respectively. The hydrochars produced only in HTC (without pretreatment) were named H-B1, H-B2, and H-B3, referring to the products derived from biomass B1, B2, and B3, respectively. When comparing HHV (Figure 1A), the H-WBs materials presented values between 20.3 and 22.0 MJ/kg, and H-Bs ranged from 11.1 to 13.1 MJ/kg. Acid wash, followed by HTC, promoted an increase of about 1.8 times compared to hydrochar derived from biomass without pretreatment. However, this increase can be attributed to acid washing since the values obtained for the H-WBs hydrochar are similar to the biomass of WBs origin (Table 1). In other words, HTC does not contribute to the increase in HHV, and the statistical difference (*p* < 0.05) between H-WBs and H-Bs reflects the ash presence’s negative effect.

**Figure 1:**
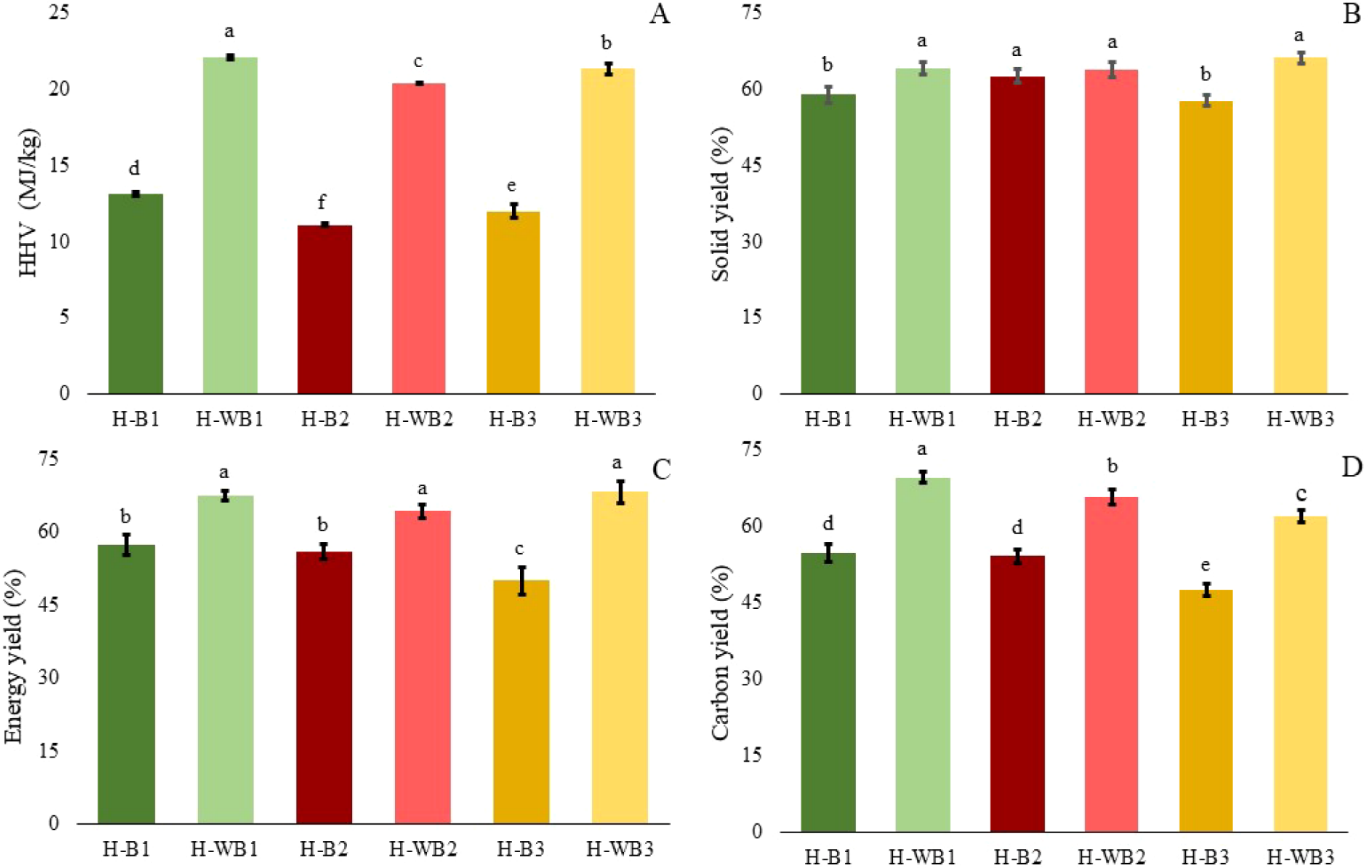
HHV (A), solid yield (B), energy yield (C), and carbon yield (D) of hydrochar produced from microalgae biomass before (H–B) and after biomass pretreatment (H-WB) (*n* = 3, vertical bars represent the standard deviation). Means that do not share a letter are significantly different.

The pretreatment provided a significant increase (*p* < 0.05) in the solids recovery for the hydrochar H-WB1 (64.0%) and H-WB3 (66.0%) compared to the raw hydrochar, i.e., H-B1(58.8%) and H-B3 (57.6%), respectively (Figure 1B). For biomass B2, the acid wash did not affect the solids yield after the HTC, with H-WB2 (63.7%) being statistically equal (*p* > 0.05) to H-B2 (62.4%). Liu and collaborators^18^ found that HTC from microalgae biomass previously treated with acid solution and lower ash content showed a solids recovery approximately 26% higher than HTC from natural biomass. The authors suggested a substantial enhancement of microalgae hydrolysis by high ash content.

Figure 1B presents the solids recovery results considering the term m_biomass_ in eq 1 (described in item 4.4) as the mass of raw biomass. At the same time, for H-WBs the reference base was the mass of pretreated biomass. The calculation was performed in this way to evaluate HTC’s performance alone. The results differ when the performance of the two treatment stages is evaluated together, i.e., acid wash + HTC (considering raw biomass as the calculation base for H-Bs and H-WBs) (Figure S1). In this case, there was a lower solids recovery in H-WBs (40.8–42.3%) compared to H-Bs (57.6–62.4%) due to the mass loss that occurred with the acid wash.

Hydrothermal torrefaction (160 and 170 °C for 5 and 10 min) with acid hydrolysis pretreatment of *C. vulgaris* biomass showed decreasing solids recovery as the acid concentration increased. The highest solids recoveries reported were 54.5, 31.0, and 23.9% at H_2_SO_4_ concentrations of 0, 0.1 M, and 0.2 M, respectively.^21^ According to the authors, when the acid hydrolysis reaction is more vigorous, more energy from the biomass is distributed to the aqueous phase.^21^

The application of acid washing reduces the ash content, resulting in a decrease in biomass mass. As a result, there is a lower recovery of solids when the acid washing + HTC process is analyzed together. In addition, during HTC, compounds are transferred from the biomass to the aqueous and gaseous phases, contributing to the loss of mass in the hydrochar. Thus, the loss of solids appears to be inherent to HTC, and the percentages obtained may vary according to the characteristics of the biomass used and the HTC conditions adopted, with percentages between 20.6% and 97.0% being observed^31^

Energy yield was calculated to complement the interpretation of the results (Figure 1C). This is an essential parameter for evaluating the performance of the processes applied in this study, as it summarizes the information on HHV and solids recovery. The hydrochar from acid-washed biomass (H-WBs) had an energy recovery of 64.2% to 68.2%, which was significantly higher (*p* < 0.05) than the hydrochar from biomass without pretreatment (H-Bs), in which the percentages varied from 50.0% to 57.2%. This behavior was maintained when the combined performance of acid washing + HTC was analyzed (Figure S1) (64.4 to 68.2%). The increase in HHV probably compensated for the mass loss during acid washing. These results demonstrate that pretreatment was a fundamental step in improving the final production of biofuels regardless of the initial composition of the biomass.

The results observed in the present study corroborate previously reported results, which used acid washing and subsequent HTC of naturally grown microalgae (*Scenedesmus*) cultivated in a pond with domestic sewage. The authors found that hydrochar produced with biomass without acid washing (ash = 44.7%) presented an energy recovery of 33.3 to 44.1%, while hydrochar derived from pretreated biomass (ash = 14.5%) reached 47.8 to 51.9%.^18^ The presence of ash did not favor energy, as discussed earlier in section 3.1, negatively influencing the HHV because it is a material that does not contribute to the combustion process.

When analyzing the carbon recovery (Figure 1D) in the hydrochar produced with raw biomass, the highest percentage was observed in H-B1 (54.7%). Using pretreated biomass, the best performance was verified in H-WB1 (69.5%). These results are consistent with the HHV values presented in Figure 1A. Comparing the carbon recovery between the H-Bs and H-WBs hydrochar, the performance was 61.9 to 69.5% for H-WBs, significantly higher (*p* < 0.05) than the percentages of 47.5–54.7% observed in H-Bs. The analysis of the acid washing + HTC steps (Figure S1) conferred a carbon recovery of 67.4 to 68.2%, reinforcing the better performance of hydrochar from pretreated biomass.

Important observations can be made when comparing Figures 1 and S1. First, HTC alone does not appear to have effectively promoted an increase in HHV in relation to the precursor biomass. This result may be due to the operating conditions adopted (170 °C for 10 min), which possibly resulted in a low degree of carbonization. Additional research with the application of higher temperatures and reaction times is necessary.

A second observation is that acid washing contributed to the increase in HHV and C (ultimate analysis, Table S2) in the hydrochars but had a negative effect on solids recovery. A balanced analysis of these advantages and disadvantages can be inferred from the recovery of energy and carbon. The results indicate that the advantages (increase in HHV and C) outweighed the negative aspects (low degree of carbonization and loss of solids), since the H-WBs hydrochars showed potential for application in energy routes. This potential occurred with low production energy consumption as operating conditions were mild.

#### 3.2.2 Nitrogen and Sulfur

The concentrations of N and S determined in all hydrochars (Table 2) were slightly lower than those initially observed in the raw biomass (Table 1). The most significant N and S reduction was observed in H-B3 with 23.0 and 11.1%, respectively. This reduction may be due to the gas losses during HTC and transfer to the aqueous fraction. Even so, the most considerable fraction of N and S present in biomass (>77%) remained in the hydrochar, indicating that these elements were in forms resistant to thermal decomposition for the HTC conditions adopted in this study.

**Table 2:** Nutrient Characterization of the Hydrochar Obtained after HTC (on a Dry Biomass Basis)^a^ ^b^

Element	H-B1	H-B2	H-B3	H-WB1	H-WB2	H-WB3
N (g/kg)	33.7 b	41.6 bc	46.5 bc	70.7 a	66.1 a	61.4 a
S (g/kg)	4.6 b	4.5 b	4.5 b	6.2 a	6.5 a	5.9 a

a H–B= refers to hydrochar from raw biomass; H-WB= refers to hydrochar from acid-washed biomass.

b The values refer to the averages (*n* = 3). Means that do not share a letter are significantly different at the 5% level.

H-WBs hydrochar showed more attractive characteristics to biofuel applications, such as higher HHV and lower ash content, providing more energy during combustion. However, the N and S concentrations in the biomass became higher after the acid wash step. They remained in the hydrochar, being significantly higher (*p* < 0.05) in the H-WBs if compared to H–B. This characteristic can limit the use of H-WBs hydrochar as biofuel due to SO_*x*_ and NO_*x*_ emissions risk. In the characterization of different coals, values below 1.5% for N and between 0.2 and 2.8% for S were observed.^32^ When comparing these values with those obtained for H-WBs hydrochar, it is possible to verify that S concentrations are similar to the coals, while N exceeds by up to 4.5 times. Furthermore, restrictions of S less than 0.5% and N less than 2.5% are applied when considering the specifications for thermally treated biomass fuels (ISO 17225).^33^ These limits were unmet, indicating the need for S and N reduction.

Nitrogen and sulfur in fuel products derived from microalgae, such as hydrochar and bio-oil, are among the main challenges for energy recovery from this biomass.^34,35^ Increasing the severity of HTC conditions may reduce nitrogen content; however, this approach involves trade-offs with product yield and energy efficiency and must be carefully optimized.^35^ An alternative strategy is the cohydrothermal carbonization (co-HTC) of nitrogen- and sulfur-rich residues with feedstocks containing lower levels of these elements. For example, co-HTC of sewage sludge with rice straw reduced up to 47% in nitrogen and 58% in sulfur contents in the hydrochar, compared to HTC of sludge alone.^36^ Nevertheless, it is essential to refine the co-HTC operational conditions, since reactions such as Maillard (between carbohydrates and proteins) and Mannich (between lignin and proteins) may enhance nitrogen retention in the solid phase.^35^

Further options to mitigate N and S include catalytic HTC and the use of nitrogen scavengers. For instance, the application of layered double oxide (LDO) catalysts based on Ni–Mg–Al during HTC of sewage sludge achieved nitrogen reductions of up to 65%, attributed to the migration of nitrogenous compounds from the solid to the aqueous phase.^37^ In addition, denitrogenation and desulfurization techniques—commonly used in fuel upgrading—such as adsorption or solvent extraction, have shown potential, although studies on their application to microalgal biofuels remain limited.^38^ The viability of these strategies must also consider economic feasibility, as additional processing steps and material inputs for N and S mitigation can increase the overall production cost of hydrochar.

The graphical analysis of N and S content versus energy yield (Figure S2) revealed a clear trade-off resulting from acid washing: while pretreatment improves fuel quality in terms of energy, it simultaneously offers environmental risk due to increased N and S. The decision to apply acid pretreatment should consider the full system performance, including potential emissions. Integrated tools such as life cycle assessment and techno-economic analysis are critical to quantify the environmental and economic implications of these trade-offs and support decision-making in biofuel production systems.

### 3.3 Van Krevelen Diagram

The Van Krevelen diagram was used to understand the pretreatment effects and HTC reactions (Figure 2). Acid wash contributed to a reduction in the O/C and H/C atomic ratios compared to raw biomass with WBs in behavior toward dehydration, except for WB1, which presented O/C ratios approximately 8% higher than B1. The hydrochar produced with raw biomass (HBs) presented a change in the H/C ratio of less than 5%. The O/C ratio increased between 4 and 12% compared to the original biomass (Bs). HTC of sewage sludge showed an increase in the O content attributed to the hydrolysis of the polysaccharides.^39^

**Figure 2:**
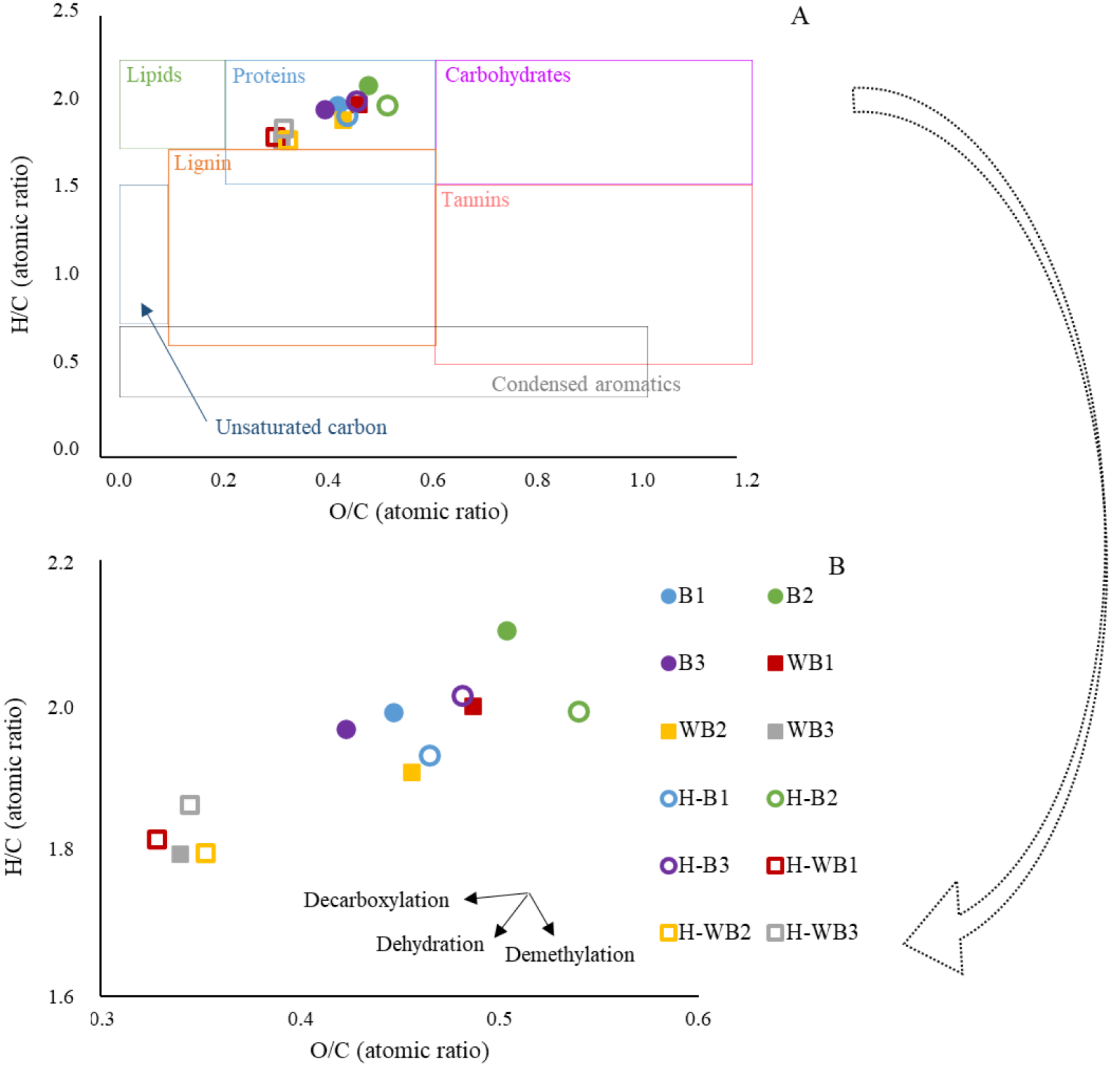
Van Krevelen diagram and the H/C and O/C atomic ratios of biomass and hydrochar (A), data amplification (B). B1, B2, B3 = raw biomass; WB1, WB2, WB3 = acid-washed biomass; H-B1, H-B2, H-B3 = hydrochars from raw biomass; and H-WB1, H-WB2, H-WB3 = hydrochars from acid-washed biomass.

More promising results were observed when relating the characteristics of the pretreated biomass to the hydrochar produced. Except for H-WB3, which remained similar to WB3, the other materials showed a decrease in the O/C ratio of 23% to 33%. The H/C ratio reduced from 6% to 9% when comparing WBs and H-WBs. It is possible that dehydration was the dominant process, followed by decarboxylation, which corroborates previous studies that also used algal biomass to produce hydrochar.^19^

Despite the more advantageous results of HWBs hydrochars, the ultimate analysis (Table S2) demonstrated a higher percentage of H in H-WBs materials (7.1–7.4%) compared to H-Bs (4.2–4.7%). This behavior was not reflected in the Van Krevelen diagram (Figure 2) because the C content in H-WBs (47.0–48.9%) was also higher (H-Bs = 25.3–29.2%), causing the H/C ratio to reduce in hydrochars derived from pretreated biomass. Still regarding the C content, it is noted that in HWBs this element corresponded to about half of the material composition, while in HBs the largest fraction was ash (44.5–48.8%).

The elemental ratios of O/C and H/C in all hydrochars ranged from 0.30 to 0.54 and from 1.8 to 2.0, respectively, which are typical for proteins^40^ and consistent with algal biomass characteristics. When comparing the different types of coal, the H/C ratio was high in all hydrochars, while the O/C ratio was equal to the lignite. The results obtained in the present study are consistent with the data reported in the literature for HTC of microalgae biomass, in which the obtained product is similar to lignite, although with a less condensed aromatic structure (H/C ≥ 0.7). The values of the H/C ratio in hydrochar derived from algal biomass range from 1.30 to 2.06,^8,41,42^ One possibility to reduce these values is dehydrogenation. The higher proportion of O and H reduces the total energy available in the fuel because there is a smaller amount of energy contained in the C–O and C–H bonds in relation to the C–C bonds.^43^ In addition, H consumption may occur for unfavorable water production, so a high H/C ratio may not translate into energy yield. Generally, a fuel with low H/C and O/C ratios is favorable because of the reduced energy loss, smoke and water vapor during combustion.^44^

### 3.4 Inorganics

The inorganic content present in the hydrochars is presented in Table 3. The hydrochars from biomass without pretreatment presented on average a concentration of P, K, Ca, Mg, Cu, Zn, Fe, Mg, Ni and Pb higher by up to 4.5 times compared to that observed in the precursor biomass, suggesting that HTC contributes to the enrichment of these elements in the hydrochars. A similar trend was observed for the materials from biomass with acid washing, except for the elements P and Ca that reduced from 3.6 to 4.0 g/kg and 5.4–8.7 g/kg in WBs to 1.9–3.0 g/kg and 0.8–0.9 g/kg in H-WBs, respectively. HTC of microalgae and digested sewage sludge demonstrated that heavy metals were retained mainly in the hydrochars. The heavy metals present in these materials may have a high boiling point and low solubility in water, which minimizes their transformation into liquid and/or gaseous products.^45^

**Table 3:** Inorganic Characterization of Hydrochars (on a Dry Biomass Basis)^a^

Parameter	H-B1	H-B2	H-B3	H-WB1	H-WB2	H-WB3
P (g/kg)	56.8	71.3	70.2	2.7	3.0	1.9
K (g/kg)	9.5	9.5	8.2	0.5	0.5	0.6
Ca (g/kg)	84.1	118.4	113.8	0.9	0.8	0.8
Mg (g/kg)	13.7	11.4	7.4	0.4	0.4	0.3
Cu (g/kg)	0.5	3.1	4.2	0.6	1.5	1
Fe (g/kg)	6.7	4.6	3.6	7.6	7.1	5.7
Zn (g/kg)	37.8	38.3	46.6	1.1	1.2	0.7
Mn (g/kg)	0.55	0.50	0.30	0.06	0.07	0.05
Na (mg/kg)	0.40	0.60	0.50	0.01	0.07	0.10
Ni (mg/kg)	8.8	7.9	7.6	11.2	11.7	14.5
Pb (mg/kg)	20.8	28.6	37.5	5.7	11.2	14.5
Cr (mg/kg)	0.5	0.3	0.28	Nd	0.3	nd

a HB1, HB2, HB3 = hydrochars from raw biomass; HWB1, HWB2, HWB3 = hydrochars from acid-washed biomass.

In general, the higher contents of inorganic compounds in the raw biomass and the action of HTC that contributed to the presence of metals in the hydrochars resulted in a considerably higher content of these elements in H-Bs than H-WBs. With emphasis on P and Ca that reduced from 56.8–71.3 g/kg and 84.1–118.4 g/kg to 1.9–3.0 g/kg and 0.8–0.9 g/kg respectively, two exceptions can be noted. First, a certain stability of Fe that maintained similar concentrations in H-Bs (3.6–6.7 g/kg) and H-WBs (5.7–7.6 g/kg). Second, the addition of Ni in the H-WBs hydrochars of up to 14.5 mg/kg, while in H-Bs the contents were lower than 7.6 mg/kg. These results indicate that acid washing followed by HTC can interfere in different ways with the metal content and make certain elements more concentrated in the hydrochars.

The concentrations of Zn, Cu and Pb observed in all hydrochars do not meet the general quality specifications required for thermally treated biomass fuels (ISO 17225), which are up to 0.1 g/kg; 0.02 g/kg and 10 mg/kg, respectively.^33^ An exception was observed for H-WB1, which presented a Pd content equal to 5.7 mg/kg. This inadequacy should be adjusted for the safer use of hydrochars with biofuels to minimize particulate emissions, recycling and disposal of residual ash. Nonetheless, the hydrochars derived from acid-pretreated biomass exhibited improved HHV and energy recovery, supporting their potential for use as bioenergy sources under regulatory adjustments.

### 3.5 Chemical and Structural Characterization of Hydrochar

The raw biomass presented similar diffractograms, indicating an amorphous characteristic with the atomic structure without order at a long distance (Figure 3). HTC, without pretreatment, did not contribute to the formation of semicrystalline structures. The acid washing provided diffraction patterns in the WB3 biomass similar to those observed in B3, while C_10_H_15_Na, C_12_H_22_O_11_, and SiO_2_ were observed in the others (WB1 and WB2). These same compounds remained after HTC, with the hydrochar H-WB3 showing the greatest structural changes, with additional peaks corresponding to SiO_2_ and NaCl. The presence of SiO_2_ after HTC of microalgae biomass has been reported previously.^8^ Silica (SiO_2_.H_2_O) integrates the microalgae cell wall and, therefore, is expected in the biomass composition and is subject to HTC reactions. In addition, SiO_2_ is insoluble in HCl^18^ and may justify its addition to the biomass and hydrochar after acid washing.

**Figure 3:**
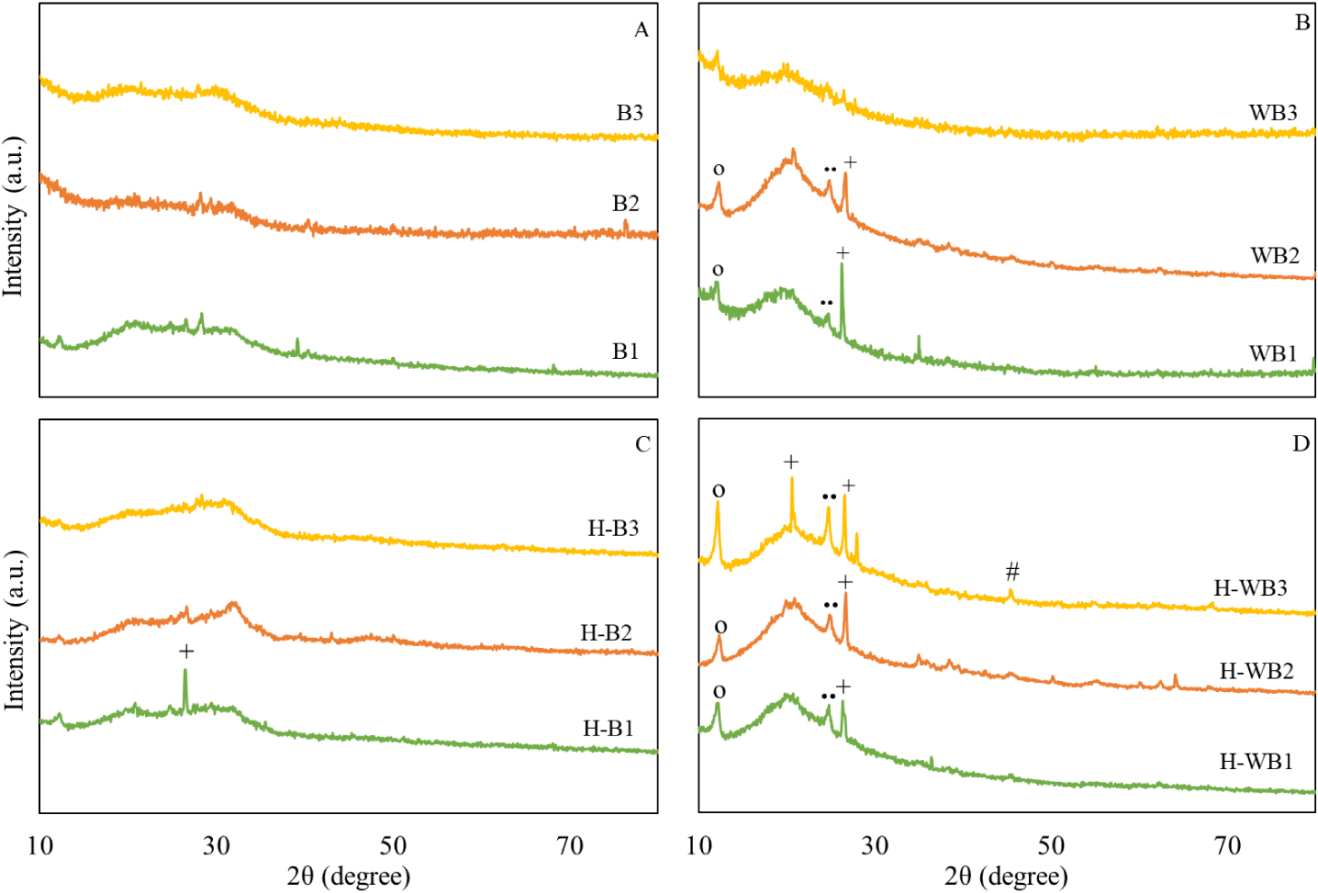
X-ray diffractograms determined on the raw biomass (B1, B2, B3) (A), after pretreatment (WB1, WB2, WB3) (B) and their respective hydrochar H-Bs (C) and H-WBs (D) ^*^CaCO_3_, ^o^C_10_H_15_Na, ^••^C_12_H_22_O_11_, ^+^SiO_2_, ^^^Fe­(PO_3_)_3_, ^x^Mg related, ^#^NaCl.

SEM was used to compare biomass and hydrochar morphology (Figures S3 and S4). Biomass without prior acid washing (Figure S3 A-C) presented a rough surface that remained after HTC. For the hydrochar obtained from these biomass samples (Figure S4 A-C), fragmented structures were also observed due to the presence of amorphous carbon.^18^ In addition, hydrolysis during HTC can reduce the size of biomass particles. The roughness may be due to the presence of ash in higher concentrations because, when analyzing the images referring to WB biomass and H-WB hydrochar (Figures S3 D-F and S4 D-F, respectively), there is a smoother surface with minimization of the tortuous surface. Despite this result, the morphology of the H-WB hydrochar was predominantly irregular. Zhang and collaborators^17^ mentioned that the high N content in microalgae biomass could provide this characteristic.

The analysis of the FT-IR spectra (Figure 4) showed that the raw and pretreated biomass presented similar spectral profiles with several vibrational modes characteristic of the investigated systems. For example, bands from 2900 to 2700 cm^–1^ are related to the symmetrical and asymmetrical stretching movements of C–H bonds of methyl and methylene groups (indication of the presence of lipids).^46^ The band around 1650 cm^–1^ is attributed to the amide I vibrational mode and at 1550 cm^–1^ to the amide II mode. They are vibrational movements involving C = O, N–H, and C–N bonds, all characterizing the proteins. The most significant differences between raw and pretreated biomass were observed in the bands between 1200 and 900 cm^–1^, which can be related to carbohydrates, and bands in the range of 600 and 560 cm^–1^, which can be attributed to the stretching vibration of P–O.^47,48^ In these intervals, pretreated biomass showed a reduction in the intensity of the bands. This result reflects biomass composition after pretreatment and can indicate carbohydrate degradation, representing a lower concentration of P in biomass with acid washing.

**Figure 4:**
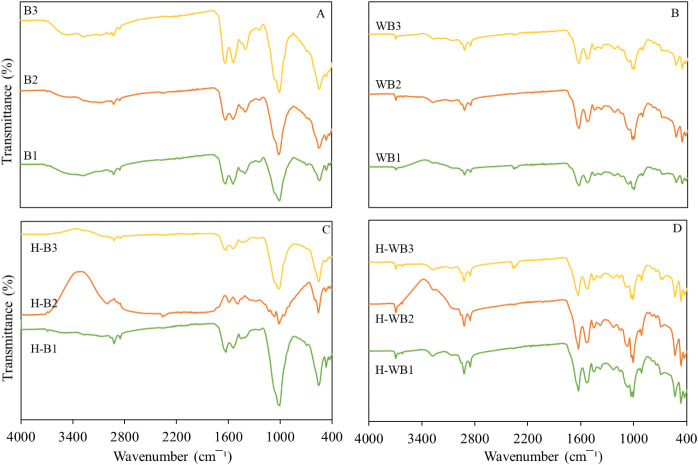
FT-IR spectra of raw biomass (A), after pretreatment (B) and their corresponding hydrochar H–B (C) and H-DB (D).

When comparing the hydrochar with (H-WBs) and without (H-Bs) acid washing pretreatment (Figure 4C,D, respectively), it is observed that the absorption spectra showed, in general, similar absorption bands as precursor biomass. The main differences were observed in the bands between 1600 and 1460 cm^–1^, in which there was an increase in intensity when comparing the spectra of samples H-WBs and H-Bs. This result may be due to the higher concentration of N observed in H-WBs. The broad adsorption band near 3400 cm^–1^ was identified in H-B2 and H-WB2, which is associated with the stretching vibration of O–H groups and reveals the presence of hydroxyl groups.^18^ This behavior suggests that the other hydrochars (H-B1, H-B3, H-WB1, and H-WB3) present greater hydrophobicity than hydrochar H-B2 and H-WB2. Hydrophobicity is important for fuel storage and handling, as this affords higher resistance to humidity.^44^

### 3.6 Aqueous Phase

The concentrations of TOC, TKN and P for the aqueous phase obtained in each HTC test are presented in Table 4. In general, the results showed similar values for all tested conditions. Due to the higher concentration of N and lower content of P in the WBs biomass in relation to Bs, a similar pattern was expected in the analysis of the aqueous phases, which was not evident. Therefore, the acid wash does not seem to affect the analyzed parameters. The same temperature and pressure conditions adopted in all tests may have contributed to this result. A previous study demonstrated that as the HTC temperature increases, the greater the dissolution of organic matter.^49^

**Table 4:** Characterization of the Aqueous Phase Obtained after HTC^a^

Parameter	H-B1	H-B2	H-B3	H-WB1	H-WB2	H-WB3
TOC (g/L)	6.8	6.7	6.9	6.6	5.3	6.0
TKN (g/L)	1.9	1.4	0.3	1.9	1.4	0.8
P (mg/L)	150.9	99.2	122.7	131.4	168.6	168.1

a HB1, HB2, HB3 = hydrochars from raw biomass; HWB1, HWB2, HWB3 = hydrochars from acid-washed biomass.

Despite similar TOC, TKN, and *p* values across all treatments, the aqueous phase retained a significant nutrient load and organic matter, particularly after HTC of biomass with high nitrogen content. These results highlight the importance of evaluating the aqueous fraction not only from a pollution perspective but also as a potential resource for biogas production (via anaerobic digestion) or nutrient recovery. However, reuse strategies must consider the presence of refractory compounds, C:N:P ratios, and possible inhibitors. Thus, more in-depth studies are needed to understand better the chemical nature and environmental behavior of the HTC process water. Such investigations should include identification of specific organics (e.g., carboxylic acids, phenols), heavy metals, and potential toxicity.

## 4 Conclusions

This study demonstrated that acid washing reduced the mass of pretreated biomass due to the solubilization of acid-soluble compounds. Although ash content decreased, the concentrations of proteins and elements such as C, N, and S increased. These compositional changes were reflected in the hydrochar, which presented improved characteristics for use as a biofuel, except for the elevated contents of nitrogen, sulfur, zinc, copper, and lead, requiring regulatory adjustments.

Therefore, acid pretreatment was found to be effective for enhancing energy and carbon recovery. However, the drawbacks of this strategy—particularly regarding the environmental impact of N and S—must not be overlooked. Future research should focus on developing specific strategies to reduce N and S retention in hydrochar and assess the trade-offs between energy yield and environmental emissions.

Moreover, the feasibility of implementing acid pretreatment in real-world applications requires integrated assessments based on thermogravimetric analysis and combustion indices (primarily), and life cycle analysis (LCA) and techno-economic analysis (TEA). These tools are essential not only for evaluating environmental and economic performance but also for identifying bottlenecks and opportunities under scale-up conditions for sustainable deployment of microalgal bioenergy technologies.

## Supplementary Material



## Notes

Additional experimental details include a table and graphs with supplementary results­(PDF)
