# Supplementary material for: Hydrothermal Carbonization of Microalgae Biomass from Wastewater Treatment: Effects of Acid Pretreatment
Source: ACS Omega. 2025 Jul 21;10(30):33138–48. doi: 10.1021/acsomega.5c02582 (PMC12332549; doi:10.1021/acsomega.5c02582)
Supplement: Supplementary file 1 [file ao5c02582_si_001.pdf]

Supporting Information

Hydrothermal carbonization of microalgae biomass from wastewater treatment: effects of acid pretreatment

Adriana Paulo de Sousa Oliveira[a], Paula Peixoto Assemany[b], Jackeline de Siqueira Castro[a*], Maurino Magno de Jesus Júnior[c], Fábio de Ávila Rodrigues[c], Luiz Fernando Cappa de Oliveira[d], Mariana Toledo Clemente Campos[d], Angélica de Cássia Oliveira Carneiro[e], Maria Lúcia Calijuri[a]

[a] Department of Civil Engineering, Federal University of Viçosa, Viçosa, 36570-900, Minas Gerais, Brazil

b Department of Environmental Engineering, Federal University of Lavras, Lavras, 37200-900, Minas Gerais, Brazil

c Department of Chemistry, Federal University of Viçosa, Viçosa, 36570-900, Minas Gerais, Brazil

d Department of Chemistry, Federal University of Juiz de Fora, Juiz de Fora, 36036-900, Minas Gerais, Brazil

e Department of Forest Engineering, Federal University of Viçosa, Viçosa, 36570-900, Minas Gerais, Brazil

* Corresponding author: jackeline.castro@ufv.br. Mailing address: Federal University of Viçosa, Viçosa Campus, Exact Sciences Center, Av. Peter Henry Rolfs, Viçosa – MG, CEP 36570-900

A preliminary test was conducted to identify the best treatment for ash removal from microalgae biomass. Eleven different solutions were tested, including washing the biomass with distilled water at room temperature, distilled water at 80°C, and different concentrations of HCl and NaOH solutions. Among these treatments, the best performance was obtained with the use of 0.25 M HCl (Table S1), which gave the biomass the lowest ash content while providing the highest carbon concentration.

Table S1: Applied treatments for ash removal and observed changes in carbon and ash contents (n=3). Means that do not share a letter are significantly different.

Treatments	Carbon (%)	Ash (%)
Raw biomass	20.6 d	39.7 b
Water	26.0 c	44.2 a
Water at 80°C	25.6 c	42.4 a
0.10M HCl	44.2 b	6.3 c
**0.25M HCl**	**49.2 a**	**3.8 e**
0.50M HCl	45.1 b	4.4 d
1.0M HCl	47.5 ab	5.1 c
4.0M HCl	45.0 b	4.4 d
0.25M NaOH	23.4 cd	37.7 b
0.50M NaOH	24.5 cd	38.8 b
1.0M NaOH	26.2 c	40.8 ab
4.0M NaOH	28.4 c	42.1 a

Table S2: Ultimate analysis of hydrochars.

Hydrochars	C	H	N	S	O	Ash
	%					
H-B1	29.2	4.7	3.1	0.5	18.1	44.5
H-B2	25.3	4.2	3.1	0.4	18.2	48.8
H-B3	26.8	4.5	3.5	0.4	17.3	47.5
H-WB1	48.9	7.4	6.7	0.7	21.4	14.8
H-WB2	47.4	7.1	6.8	0.7	22.3	15.6
H-WB3	47.0	7.3	7.2	0.7	21.6	16.2

H-B1, H-B2, H-B3 = hydrochars from raw biomass; and H-WB1, H-WB2, H-WB3 = hydrochars from acid-washed biomass.

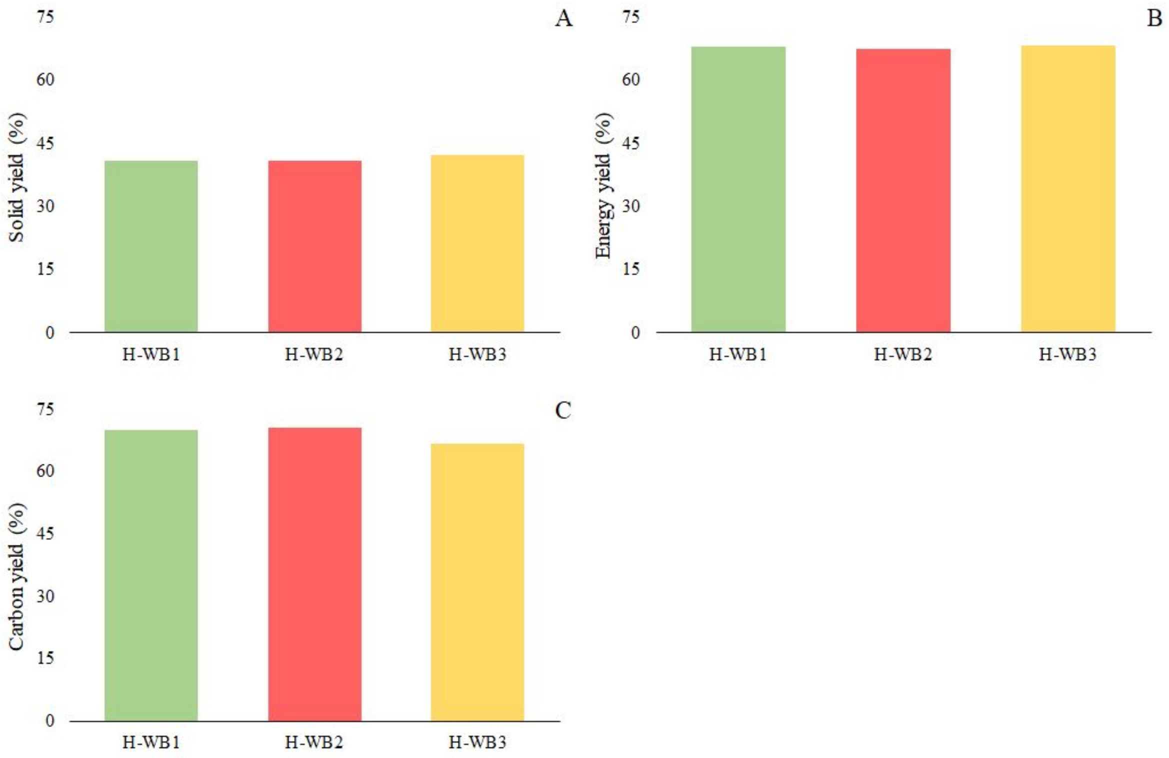

Figure S1: Solid yield (A), energy yield (B), and carbon yield (C) of hydrochar produced from microalgae biomass after biomass pretreatment considering raw biomass as the calculation base.

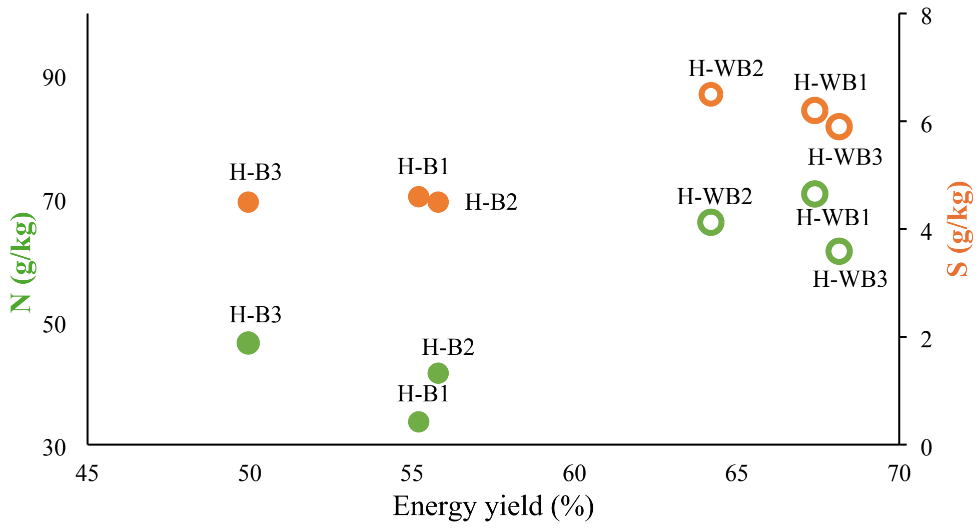

Figure S2: Trade-off between energy yield and nitrogen and sulfur contents in hydrochars produced from raw (H-B) and acid-washed (H-WB) microalgal biomass. Green circles refer to nitrogen concentrations and orange circles refer to sulfur concentrations.

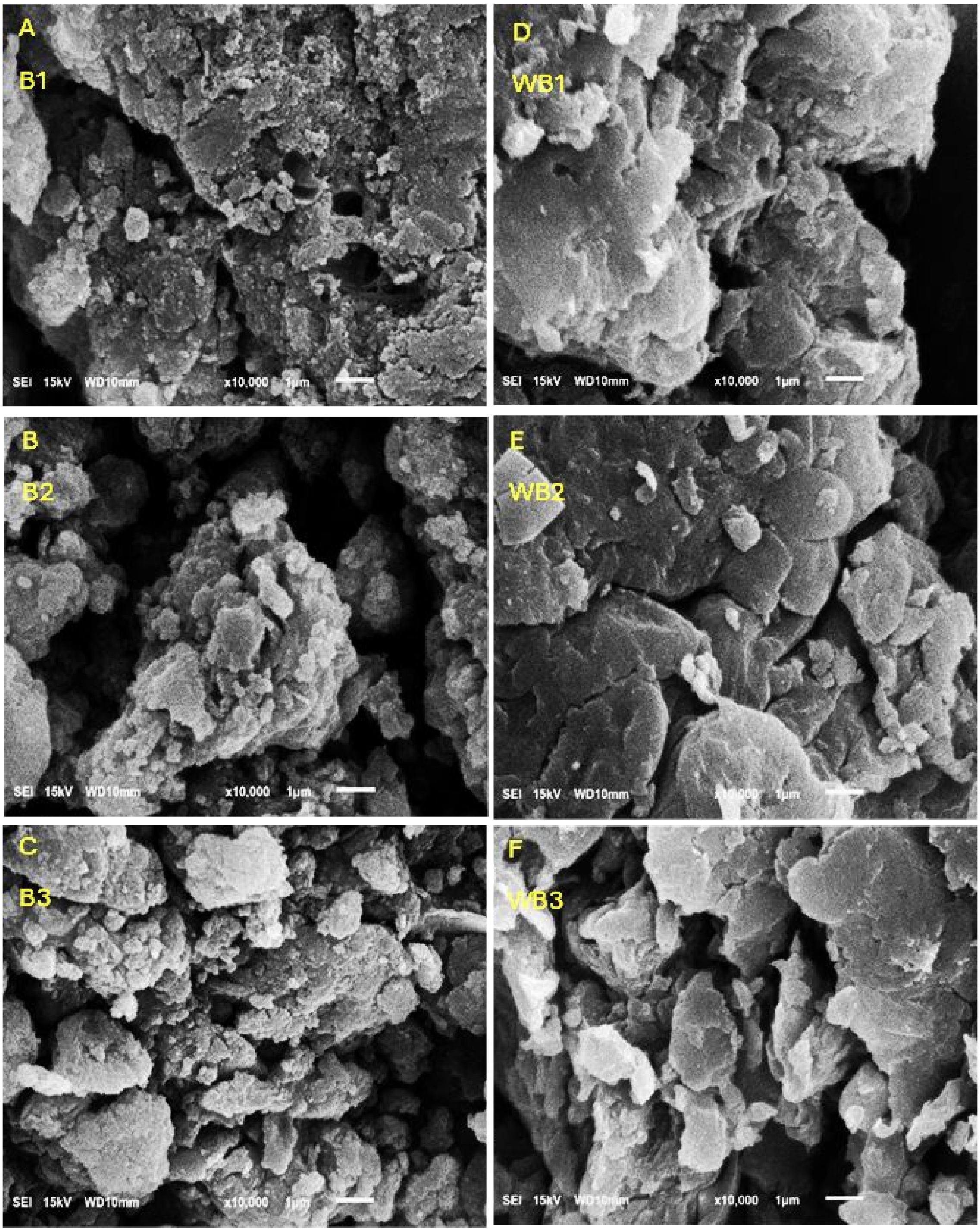

Figure S3: Scanning electron microscopy obtained for the raw biomass (A-C), and after acid washing (D-F).

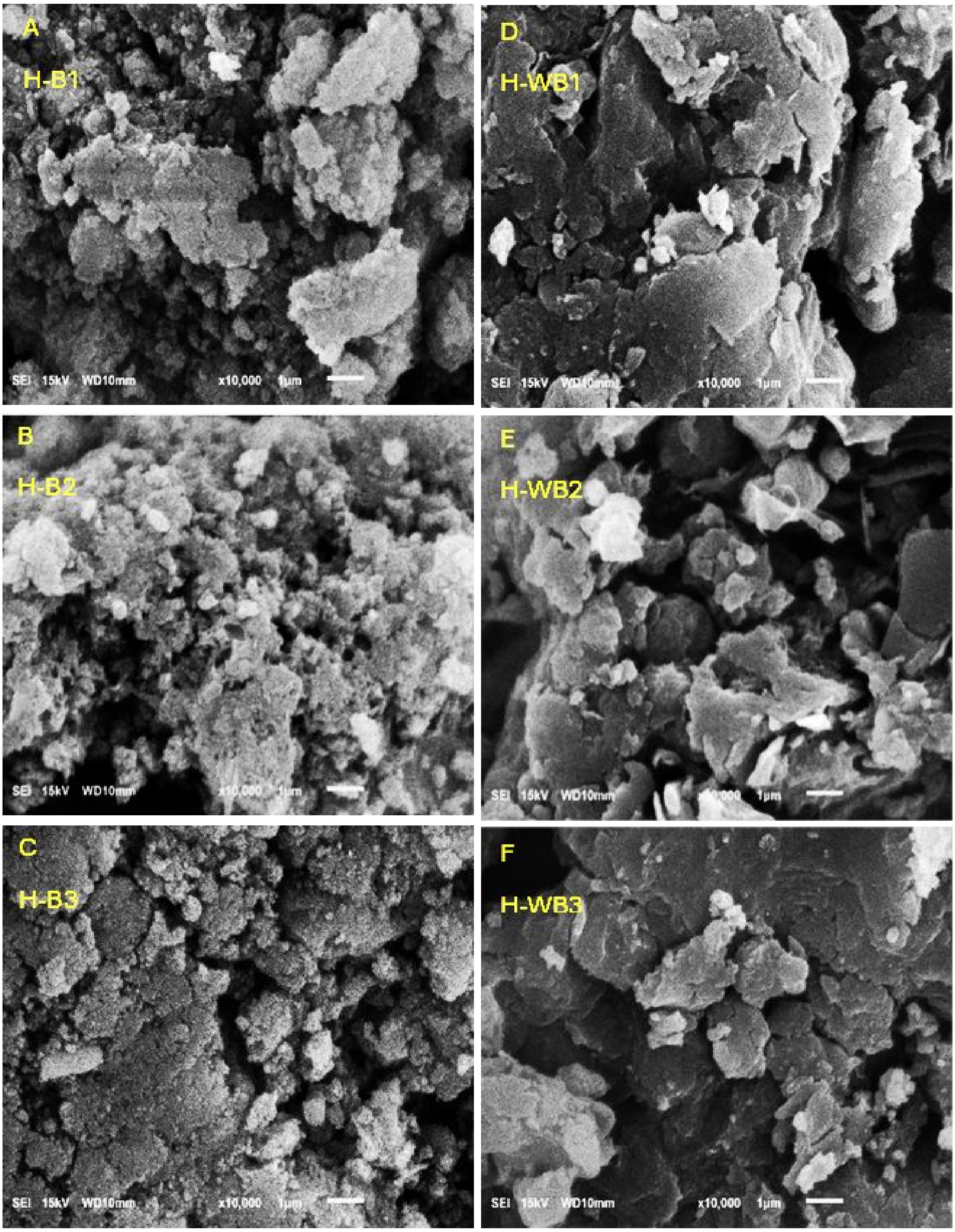

Figure S4: Scanning electron microscopy obtained for the hydrochars from raw biomass (A-C), and pretreated biomass (D-F).
